# LTBP1 promotes esophageal squamous cell carcinoma progression through epithelial-mesenchymal transition and cancer-associated fibroblasts transformation

**DOI:** 10.1186/s12967-020-02310-2

**Published:** 2020-03-26

**Authors:** Rui Cai, Ping Wang, Xin Zhao, Xiansheng Lu, Ruxia Deng, Xiaoyu Wang, Zhaoji Su, Chang Hong, Jie Lin

**Affiliations:** 1 Department of Pathology, Nanfang Hospital, Southern Medical University, Guangzhou, Guangdong Province People’s Republic of China; 2 Department of Pathology, School of Basic Medical Sciences, Southern Medical University, Guangzhou, Guangdong Province People’s Republic of China; 3 Guangdong Province Key Laboratory of Molecular Tumor Pathology, Guangzhou, Guangdong province People’s Republic of China

**Keywords:** LTBP1, Epithelial–mesenchymal transition, FN1, Cancer-associated fibroblasts, Metastasis, Esophageal squamous cell carcinoma, Carcinogenesis

## Abstract

**Background:**

Esophageal squamous cell carcinoma (ESCC) is one of the most prevalent cancers worldwide. Due to its high morbidity and mortality rates, it is urgent to find a molecular target that contributes to esophageal carcinogenesis and progression. In this research, we aimed to investigate the functions of Latent transforming growth factor β binding protein 1(LTBP1) in ESCC progression and elucidate the underlying mechanisms.

**Methods:**

The tandem mass tag-based quantitative proteomic approach was applied to screen the differentially expressed proteins (DEPs) between 3 cases of ESCC tumor samples and paired normal tissues. Then the DEPs were validated in human ESCC tissues using western blot assays and GEPIA database respectively. The expression level of LTBP1 was detected in 152 cases of ESCC tissues and paired normal tissues. Loss-of-function assays were performed to detect the function of LTBP1 in vivo and in vitro. Immunofluorescence and Western blot assays were used to detect the expression of apoptosis, epithelial–mesenchymal transition (EMT) and cancer-associated fibroblasts (CAFs) markers.

**Results:**

A total of 39 proteins were screened to be up-regulated (ratio > 2.0) in all three ESCC tissues. The results of immunohistochemistry assays indicated that the expression level of LTBP1 was higher in ESCC tissues than that in paired normal tissues (p < 0.001). Overexpression of LTBP1 was positively associated with lymphatic metastasis in ESCC (p = 0.002). Down-regulation of LTBP1 inhibited the invasion and migration as well as metastatic abilities in vitro and in vivo. It was also observed the down-regulation of LTBP1 not only decreased the mesenchymal phenotypes but also inhibited TGFβ-induced EMT in ESCC cells. We further found that down-regulation of LTBP1 enhanced ESCC cells’ sensitivity to 5-FU treatment. Inhibition of LTBP1 expression could also attenuate induction of CAFs transformation and restrain fibroblast express fibronectin (FN1) in ESCC cells.

**Conclusion:**

Overexpression of LTBP1 was associated with lymph node metastasis in ESCC. Our results indicated that LTBP1 not only increased the malignant behaviors of ESCC cells but also induced EMT and CAFs transformation. Our studies suggested an oncogenic role of LTBP1 in ESCC progression and it may serve as a potential therapeutic target for ESCC patients.

## Background

Esophageal carcinoma (ESCA) is one of the most prevalent cancers worldwide. It ranks the 7th in terms of incidence and the 6th in mortality of all cancers [1]. There are two most common histologic subtypes: squamous cell carcinoma and adenocarcinoma. More than 375 thousand people died from esophageal cancer in China every year [2], and the overall 5-year survival rate ranges from 15% to 25% [3]. Poor prognoses of esophageal cancer are attributed to early metastasis [4–6] and chemotherapy resistance [7, 8]. Thus, it is very urgent to find more sensitive and specific molecular targets for ESCC diagnosis and treatment.

We used the tandem mass tag (TMT)-based quantitative proteomic approach to screen the differentially expressed proteins between esophageal squamous cell carcinoma (ESCC) tissues and paired normal tissues. Latent transforming growth factor β binding protein 1 (LTBP1) was one of the up-regulated proteins that was observed in ESCC tissues. LTBP1 belongs to LTBPs family. LTBPs are discovered as part of a large latent complex with TGFβ, and appear to associate with fibrillin, thus play an important role in regulation of mesenchymal cell functions [9]. LTBPs play important roles in carcinogenesis and progression [10–12].

LTBP1 is located on the chromosomal band 2p22.3, and take part in the assembly and secretion of the latent TGFβ1 [13]. LTBP1 elimination seems to attenuate fibrogenesis in hepatic stellate cells [14]. It has been reported that LTBP1 modulates cardiovascular development and is involved in the keratinization of oral epithelium, female fertility and cerebral autosomal recessive arteriopathy [15–18].

LTBP1 also plays an important role in tumorigenesis. However, the exact functions of LTBP1 seem paradoxical in various cancer types [19–23]. In ovarian cancer, LTBP1 has been reported as a potential biomarker [20]. It is an important modulator of TGF-β activation in breast cancer and malignant glioma cells [19, 21]. LTBP1 is associated with breast cancer progression and regulated by the WNT/Ca2+–CaMKII–NF-κB axis [21]. Silence of LTBP1 reduces TGF-β activity and Smad2 phosphorylation without affecting TGF-β protein levels in malignant glioma cells [19]. Elevation of LTBP1 appears to play a role in enhancing metastatic behavior in breast cancer [23]. However, LTBP1 are decreased in malignant liver tissues compared with normal liver [22].

So far, the actual role of LTBP1 in ESCC is still not fully understood. In this study, we want to elucidate the function of LTBP1 in esophageal carcinogenesis and development, and further explore the underlying mechanisms of LTBP1 in ESCC metastasis and chemotherapy resistance.

## Methods

### Tissue samples and cell culture

Human ESCC and adjacent normal tissues used in the present study were obtained from the Southern Medical University Affiliated Nanfang Hospital (Guangzhou, China). The use of human materials was approved by the Medical Ethical Committee of Nanfang Hospital. Pathological TNM staging assessed according to the American Joint Committee on Cancer (AJCC). 152 patients were recruited, including 91 (59.9%) males and 61 females (40.1%). The cell lines HET-1A, ECA109, KYSE510, HF and HUVEC were donated by the Central Laboratory of Southern Medical University Affiliated Nanfang Hospital and grown in high glucose Dulbecco’s modified Eagle’s medium (DMEM) with 10% fetal bovine serum (FBS) (both from Gibco), augmented with 1% penicillin and streptomycin. Cells were maintained in an atmosphere of 5% CO_2_ at 37 °C.

### TMT labeling and MS analysis

Samples of three ESCC tumor and corresponding normal tissues have been collected. Samples were first sonicated on ice using a high intensity ultrasonic processor in lysis buffer. The protein concentration was determined by 2-D Quant kit according to the manufacturer’s instructions. Amount of protein(100 μg) from each sample was digested with trypsin for the following experiments. The protein samples were labeled with the TMT tags and then fractionated by high pH reverse-phase HPLC using Agilent 300Extend C18 column. Peptides were dissolved with 0.1% formic acid and then directly loaded onto a reversed-phase pre-column (Acclaim PepMap 100, Thermo Scientific). The separation of peptide was performed using a reversed-phase analytical column. The resulting peptides were then analyzed by Q ExactiveTM plus hybrid quadrupole-Orbitrap mass spectrometer (ThermoFisher Scientific). The peptides were subjected to NSI source followed by tandem mass spectrometry (MS/MS) in Q ExactiveTM plus (Thermo) coupled online to the UPLC. For MS scans, the m/z scan range was 350 to 1800. Fixed first mass was set as 100 m/z.

### Immunohistochemistry and immunofluorescence

The expression of protein in 152 ESCC tissues was detected using an immunoperoxidase method. IHC staining was performed according to standard protocols as previously described [24]. The slides were incubated in primary antibody (LTBP1(1:1000, #26855; Proteintech), FN1 (1:2000, #66042; Proteintech), E-Cadherin (1:200, #3195; Cell Signaling) and TGFβ1(1:60, #ab92486, Abcam) and followed by treatment with secondary antibody and a DAB staining kit (Gene Tech #GK500705). IHC intensity for each tissue section was evaluated independently by 2 experienced pathologists who were blinded to patients’ clinical data.

Cells were incubated in primary antibodies α-SMA (1:100, #14395; Proteintech) and FN1 (1:100, #66042; Proteintech). The cells were washed with PBS for three times and followed by treatment with CoraLite488 and CoraLite594 labeled IgG (Proteintech) for an hour. After washing the cells for four times, cells were stained by DAPI (Sigma).

### qRT-PCR assays

Total RNA was isolated from ESCC tissues and cells by using a Trizol Kit. cDNA was amplified by using a first-strand cDNA synthesis kit (both from Takara). The sequences of primers were shown as followings: for GAPDH, forward 5′-GTGTCGCTGTTGAAGTCAGAG-3′, reverse 5′-CATCAAGAAGGTGGTGAAGCAG-3′, for LTBP1, forward 5′-CCGCATCAAGGTGGTCTTTAC-3′, reverse 5′-GTGGTGGTGTTCCCCTTCTC-3′.

### Western blot analysis

The proteins were separated by 10% SDS-PAGE and transferred to PVDF Membranes (Millipore). The membranes were blocked in 5% BSA for 1 h at room temperature. The membranes were incubated with primary antibodies overnight at 4 °C, GAPDH(1:5000, #10494; Proteintech), β-tubulin(1:2000, #10068; Proteintech), LTBP1(1:500, #26855; Proteintech) and FN1(1:500, #66042; Proteintech), PLOD2 (1:500, #21214; Proteintech), THBS1(1:500, #bs-2715R; Bioss), RCN3(1:500, #ab204178; abcam), α-SMA (1:1000, #14395; Proteintech), Vimentin (1:1000, #5741; Cell Signaling), N-Cadherin (1:1000,#13116; Cell Signaling), E-Cadherin (1:1000, #3195; Cell Signaling), BCL2 (1:1000, #4223; Cell Signaling), BAX (1:1000, #5023; Cell Signaling). The membranes were washed thrice with TBST and incubated with HRP-conjugated secondary antibody (1:10000) 1 h at room temperature. The membranes were washed thrice with TBST and visualized using ECL (Thermo Scientific). The relative density was quantified by ImageJ.

### Transwell migration and invasion assay

Transwell chambers were used to investigate cell migration and invasion. For migration cells were divided into si-NC and si-LTBP1 groups. After digestion and centrifugation, the cells were resuspended in serum-free medium. 100 µL cell suspension at the density of 3 × 10^5^ cells/ml was placed in the upper chamber and 600 µL DMEM containing 15% FBS was added in the lower chamber. For invasion, 2 mg/ml Matrigel (Corning) was added to the polycarbonate membrane. The cells were fixed in 4% polyformaldehyde and stained with a 0.5% crystal violet solution. Cells were observed under a light microscope, three randomly selected fields of view were counted, and photographed. Each sample was analyzed in triplicate.

### Cell proliferation assay

ECA109 and KYSE510 cells were transfected with LTBP1 siRNA or control siRNA. Cells were seeded in 96-well plates at a density of 1 × 10^3^ cells/well and cultured in DMEM with 10% FBS for 1, 3, 5, and 7 days at 37 °C. The CCK8 assay was performed as follows: cells were incubated with 100 μl cck-8 (1:10 dilution) at 37 °C for 1 h. The optical density at 450 nm was measured. Each sample was analyzed in triplicate.

### Apoptosis assay

Cell apoptosis detected by Annexin V, 633 Apoptosis Detection Kit (DOJINDO), performed as follows: the cells were collected and washed thrice with PBS as instructed, followed by staining with Annexin V/PI for 30 min at room temperature. Each sample was analyzed in triplicate.

### Enzyme linked immunosorbent assay (ELISA)

The levels of FN1 in conditioned medium of various cells were measured using Human Fibronectin ELISA Kit (CSB-E04551h; CUSABIO) according to the manufacturer’s instructions. Each sample was analyzed in triplicate.

### Indirect co-culture

For indirect co-cultures, two chambers were separated by a polycarbonate membrane with 0.4-μm pores. Fibroblasts were seeded in the lower chamber and the ESCC cells ± LTBP1 siRNA pre-treatment in the upper chamber. These cells were co-cultured for 96 h. Fibroblasts alone were used as controls.

### Tail vein metastasis assay

We performed a nude mouse tail vein transfer assay to observe tumor metastasis in mice in vivo. 4-week-old female nude mice were randomly divided into two groups (ECA109 shLTBP1, ECA109 CON) and injected 200 µl cell suspension at a concentration of 2 × 10^7^ cells/ml into the tail veins. 4-weeks after injection, the mice were sacrificed, and their lungs fixed in 4% polyformaldehyde, sectioned for HE staining. Use of animal was approved by the Use Committee for Animal Care and performed in accordance with institutional guidelines.

### Statistical analysis

Data are presented as the mean ± SD. Correlation between the differential classification and LTBP1 were examined by Chi square test. For cell line studies, one-way ANOVA and paired-samples *t* test were used to compare protein levels, cell proliferation ability, apoptosis rate and migration number between different cell groups. Statistical analyses were performed using SPSS version 23.0 for Windows (SPSS Inc, USA). A threshold value was set at 0.05 (two-tailed).

## Results

### Differentially expressed proteins were screened between ESCC tissues and paired normal tissues by TMT method

**Fig. 1:**
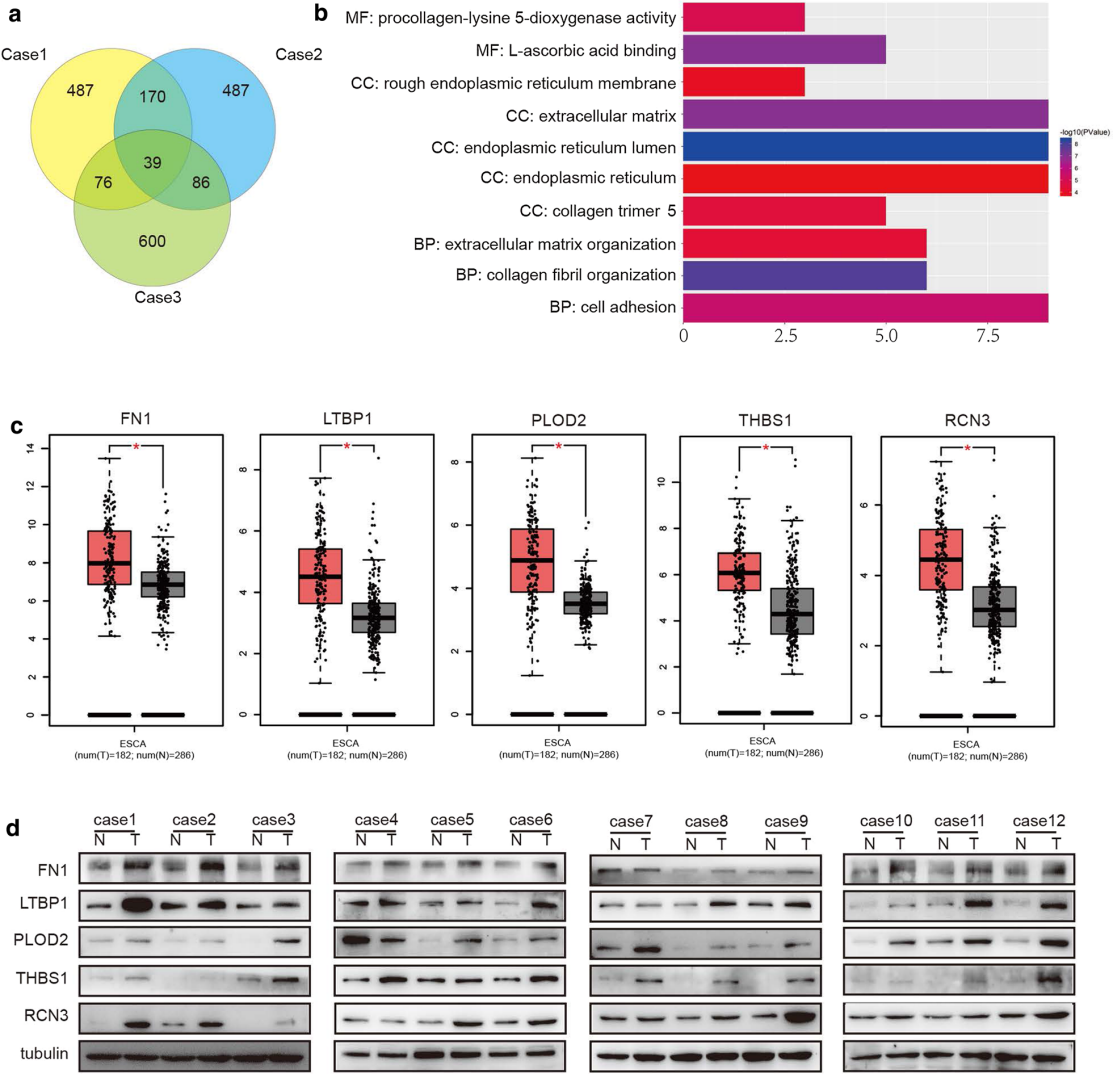
Identification and verification of DEPs in ESCC tissues. **a** Paired ESCC and adjacent normal esophageal tissues from three patients were analyzed by TMT. Twofold change was defined as the threshold for a significant change in expression. We detected 39 proteins up-regulated in cancer tissues compared with that in adjacent normal tissues (paired-samples t-test, p < 0.001). **b** Gene ontology enrichment analysis of identified DEPs which were classified into cellular component, molecular function, and biological process. **c**, **d** Validation of DEPs in ESCA. The expression of FN1, LTBP1, PLOD2, THBS1 and RCN3 were confirmed by GEPIA and western blot analysis. *N* non-tumorous tissues, *T* tumorous tissues. (one-way ANOVA and paired-samples t-test, *p < 0.05)

The TMT technology was used to explore the DEPs between three ESCC tumor samples and adjacent normal tissues. A twofold change cutoff value was used for all TMT ratios (ratio > 2.0). A total of 39 proteins were identified to be up-regulated in all three tumor tissues (Fig. 1a). To characterize the functions of the above up-regulated proteins, DAVID database [25] (https://david.ncifcrf.gov/) was used for GO enrichment analysis of molecular function (MF), biological process (BP) and cellular component (CC). The functions of DEPs were mainly involved in endoplasmic reticulum lumen, collagen fibril organization, L-ascorbic acid binding, extracellular matrix and cell adhesion (Fig. 1b). We then randomly chose 5 up-regulated proteins, including FN1, LTBP1, Procollagen-lysine,2-oxoglutarate 5-dioxygenase (PLOD2), Thrombospondin 1(THBS1) and Reticulocalbin 3(RCN3). And we further analyzed their expression by GEPIA (Gene Expression Profiling Interactive Analysis) dataset (http://gepia.cancer-pku.cn/) [26] (Fig. 1c) and western blot analyses (Fig. 1d). Western blot results showed that FN1(11/12),LTBP1(11/12), PLOD2(11/12), RCN3(10/12), THBS1(11/12) were increased in tumor tissues (Paired-samples t-test, p < 0.05), indicating that the screening results of TMT technology were credible.

### LTBP1 was up-regulated in ESCC and associated with lymphatic metastasis

**Fig. 2:**
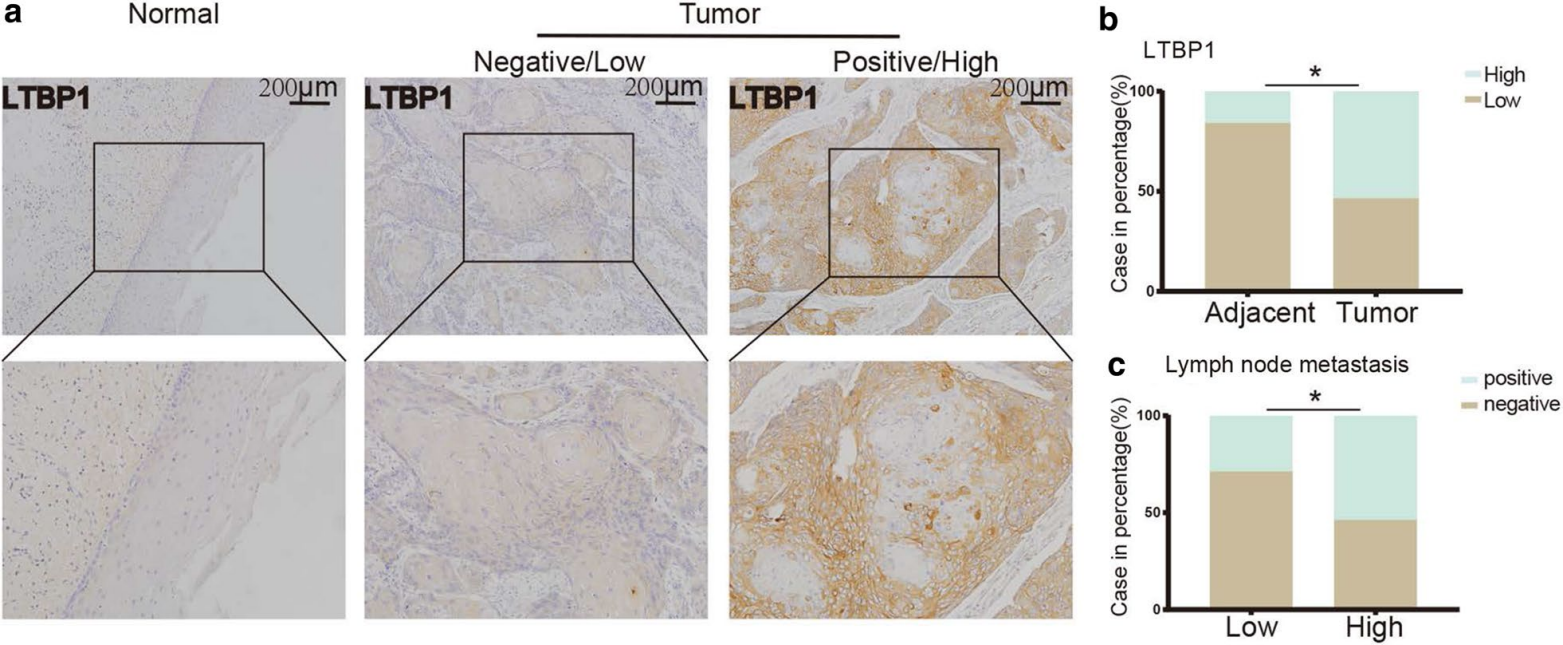
LTBP1 was up-regulated in ESCC and associated with lymphatic metastasis. **a** IHC staining of LTBP1 protein in ESCC and adjacent normal esophageal tissues. LTBP1 was localized in cytoplasm of cancer cells, but rarely expressed in normal esophageal tissues. **b** Frequency of negative, positive LTBP1 expression in ESCC and adjacent normal esophageal tissue. **c** Frequency of low/high LTBP1 expression in ESCC when categorized by lymphatic metastasis (Chi Square, *p < 0.05)

The expression of LTBP1 protein was detected in 152 cases of ESCC specimens and paired normal tissues using the immunohistochemical assay and its clinicopathological significance was further analyzed (Fig. 2a). We found that LTBP1 was predominantly located in cell cytoplasm. Among the 152 ESCC tissue samples, LTBP1 was highly expressed in 82 cases (53.9%) and was lowly expressed in the other 70 cases (46.1%) (Fig. 2b). While in the paired normal esophageal samples, the expression of LTBP1 was high in 24 (15.8%) cases and low in 128 (84.2%) cases (Fig. 2b). The above results indicated that the expression level of LTBP1 protein was significantly higher in ESCC tissues than that in normal tissues (p < 0.001, Chi Square). We further found that the expression level of LTBP1 protein was increased in ESCC with lymph node metastasis compared with ESCC without lymph node metastasis, suggesting that the expression of LTBP1 protein was positively correlated with lymph node metastasis (p < 0.05, Chi Square) (Fig. 2c, Table 1). However, no relationship was found between the expression of LTBP1 and patients’ gender, age, smoking status, drinking status, cancer family history and tumor size (p > 0.05, Chi Square) (Table 1).

**Table 1:** The relationship between LTBP1 expression and clinicopathological features in ESCC patients (n = 152)

Clinical data	Number	LTBP1 expression		χ^2^	p value
		Low	High		
Gender					
Male	91	43	48	0.131	0.717
Female	61	27	34		
Age(year)					
≥ 60	73	33	40	0.041	0.84
< 60	79	37	42		
Smoke					
Yes	76	36	40	0.106	0.745
No	76	34	42		
Drink					
Yes	64	28	36	0.236	0.627
No	88	42	46		
Family history					
Yes	16	9	7	0.748	0.387
No	136	61	75		
Tumor size (cm)					
≥ 4	61	25	36	1.054	0.305
< 4	91	45	46		
T-stage					
T1/T2	51	25	26	0.272	0.602
T3/T4	101	45	56		
Lymph node metastasis					
Positive	64	20	44	9.752	0.002*
Negative	88	50	38		
Tumor-stage					
I/II	84	44	40	3.027	0.082
III/IV	68	26	42		

* Significant difference

### Knock-down of LTBP1 inhibited migration/invasion and metastasis of esophageal cancer cells

**Fig. 3:**
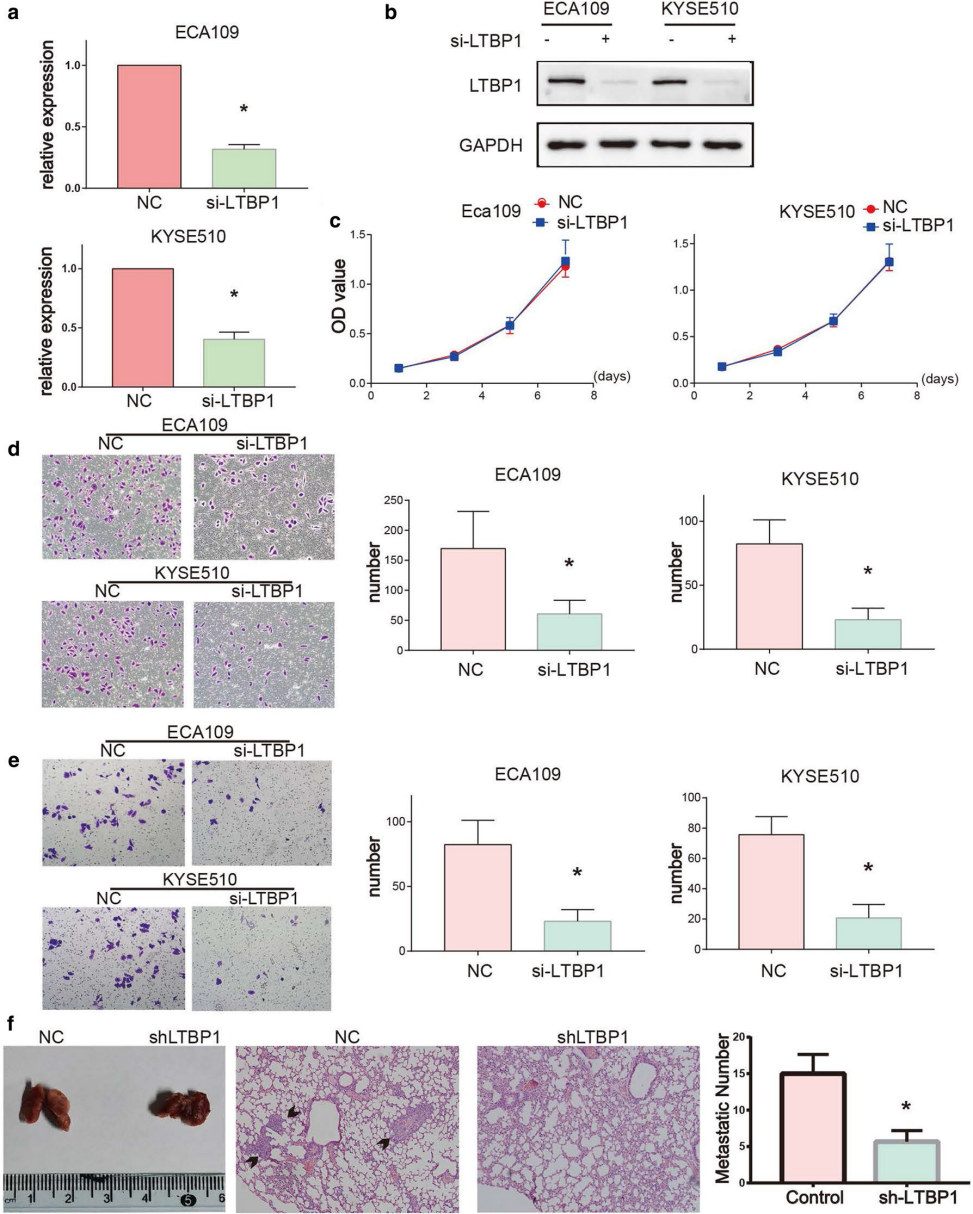
LTBP1 promoted ESCC cells migration, invasion and metastasis. **a**, **b** Efficiency of si-LTBP1 measured with qRT-PCR and Western blot. LTBP1 was reduced in si-LTBP1 groups compared with that in si-NC controls. **c** Cell proliferation capacity showed no significant changes in si-LTBP1 cells compared with the control cells, as measured by CCK8 assays. **d** Transwell migration assays of si-NC cells and si-LTBP1 cells (left). The number of cells that migrated after 24 h was counted in five randomly selected microscopic fields (right). **e** Transwell matrigel invasion assays of siNC cells and si-LTBP1 cells (left). The number of cells that invaded after 24 h was counted in five randomly selected microscopic fields (right). **f** ShLTBP1-ECA109, CON-ECA109 cells were injected into the tail vein of nude mouse. Lung tissues were autopsied 4 weeks after injection. Images of lung (left) and H&E staining (middle) of lung tissue sections were shown. Number of lung metastatic nodules (right) were significantly decreased in shLTBP1-ECA109 group. Bar graphs represent quantitative data from three independent experiments. Paired-samples t-test, *p < 0.05

We inhibited LTBP1 expression with siRNA in ECA109 and KYSE510 cells and the silencing efficiency was evaluated using qPCR and Western blot (Fig. 3a, b). The results showed that there was no significantly difference in the proliferative ability between LTBP1-inhibited cells and the control cells as detected by CCK8 assays (Fig. 3c). However, we found that LTBP1-inhibited cells showed reduced migration and invasion abilities when compared to the control cells as detected by transwell assays (Fig. 3d, e), suggesting that LTBP1 knockdown attenuated the migration and invasion of ESCC cells. We then conducted a tail vein metastasis assay to study whether LTBP1 plays an important role in tumor metastasis in vivo. The number of lung pulmonary metastatic foci was 15 ± 2.65 and 5.67 ± 1.53 in the control and shLTBP1 groups respectively (*p < 0.05, one-way ANOVA), indicating that LTBP1 knockdown reduced the metastasis capacity of ESCC cells in vivo (Fig. 3f). All the above results suggested that LTBP1 may promote ESCC cell migration, invasion and metastatic abilities in vivo and in vitro.

### Down-regulation of LTBP1 suppressed TGFβ-induced EMT

**Fig. 4:**
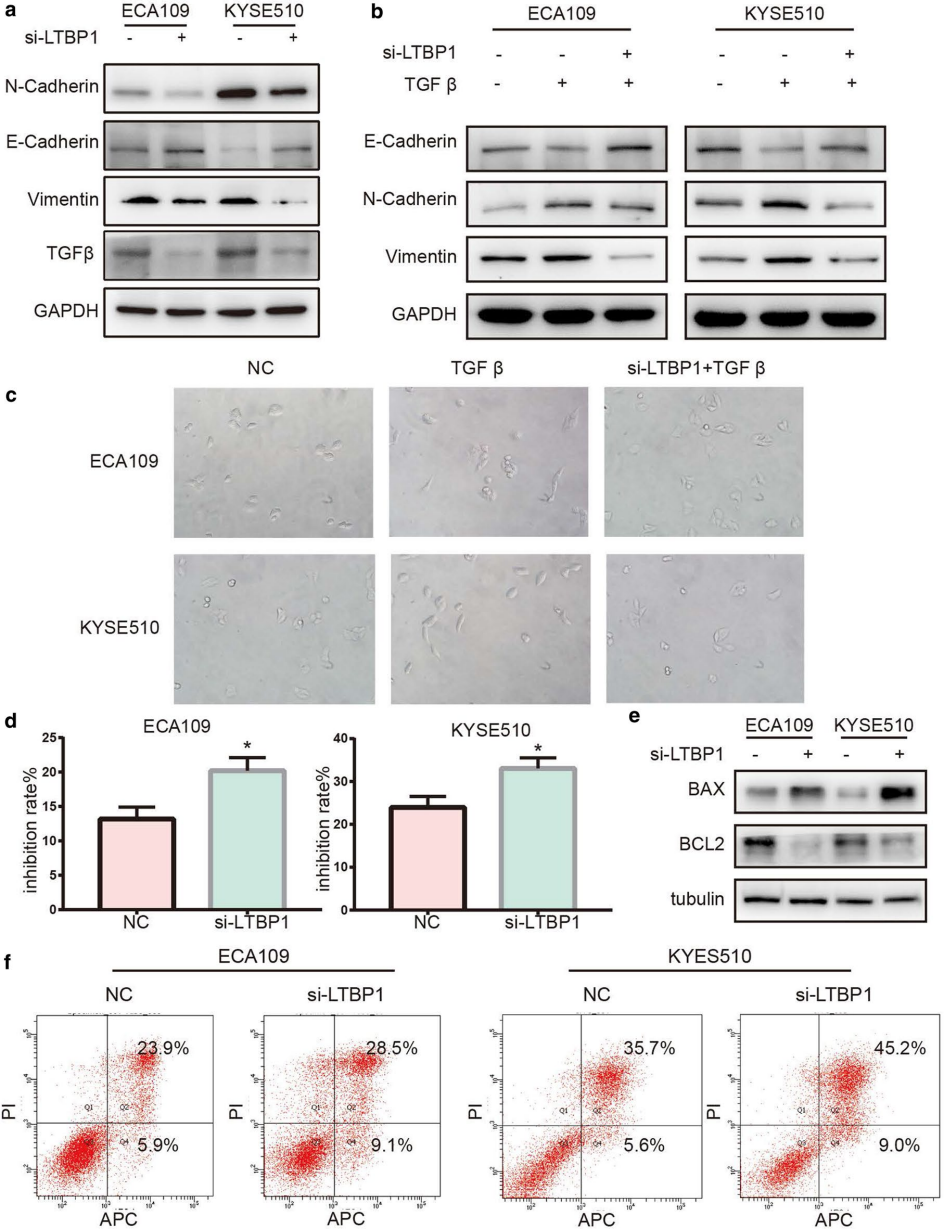
LTBP1 promoted EMT and chemoresistant in ESCC cells. **a** Silencing LTBP1 inhibited hallmarks of the EMT, including loss of N-cadherin/vimentin and accumulation of E-cadherin in ESCC cells. **b** ESCC treated with si-NC or si-LTBP1 for 24 h, and treated with/without 20 ng/ml TGFβ for an additional 48 h. Western blot analysis of EMT markers in ESCC cells. **c** Morphological changes were observed under distinct treatment conditions. **e** BCL2 expression decreased and BAX expression increased over time in si-LTBP1 cells treated with 5-FU for 48 h. **d**, **f** Inhibition rate were detected by CCK8 assays and apoptosis rate was detected by flow cytometric analysis of ESCC cells after treatment with 5-FU for 48 h. Each experiment was performed in triplicate. Paired-samples t-test, *p < 0.05

We detected the expression of EMT markers in ESCC cell lines. The results showed that the expression levels of TGFβ1, N-cadherin and vimentin were decreased while E-cadherin was increased in the LTBP1-inhibited cells as compared to the control cells (Fig. 4a), suggesting that downregulation of LTBP1 may cause mesenchymal- epithelial transition in ESCC cell lines. We then treated ECA109-siLTBP1 and KYSE510-siLTBP1 cells with TGFβ. After TGFβ treatment, the mesenchymal marker of N-cadherin and vimentin were significantly upregulated and the epithelial marker E-cadherin was downregulated (Fig. 4b). Meanwhile, the ESCC cells acquired a spindle-shaped mesenchymal phenotype (Fig. 4c). SiLTBP1 could reverse the change of EMT markers and cell phenotype induced by TGFβ1, suggesting that down-regulation of LTBP1 may suppress TGFβ-induced EMT processes. Thus, inhibition of LTBP1 may be a potential pathway to suppress EMT of tumor cells.

### Down-regulation of LTBP1 increased sensitivity to 5-Fu

We further investigated whether LTBP1 involved in the cellular sensitivity to 5-Fu treatment in ESCC cells. CCK-8 assays were performed to detect the cell viability. Compared with the control group, knockdown of LTBP1 increased inhibition rate induced by 5-Fu (Fig. 4d). In addition, we analyzed cellular apoptosis by Western blot and flow cytometry assays. After treated with 5-Fu, we found that the expression of apoptotic markers BCL2 was significantly downregulated while BAX was upregulated in LTBP1-inhibited cells (Fig. 4e). Meanwhile apoptosis rate was significantly increased in LTBP1-inhibited cells (Fig. 4f). These results suggested that downregulation of LTBP1 enhanced apoptosis induced by 5-Fu in ESCC cells.

### LTBP1 contribute to CAFs transformation induced by ESCC cells

**Fig. 5:**
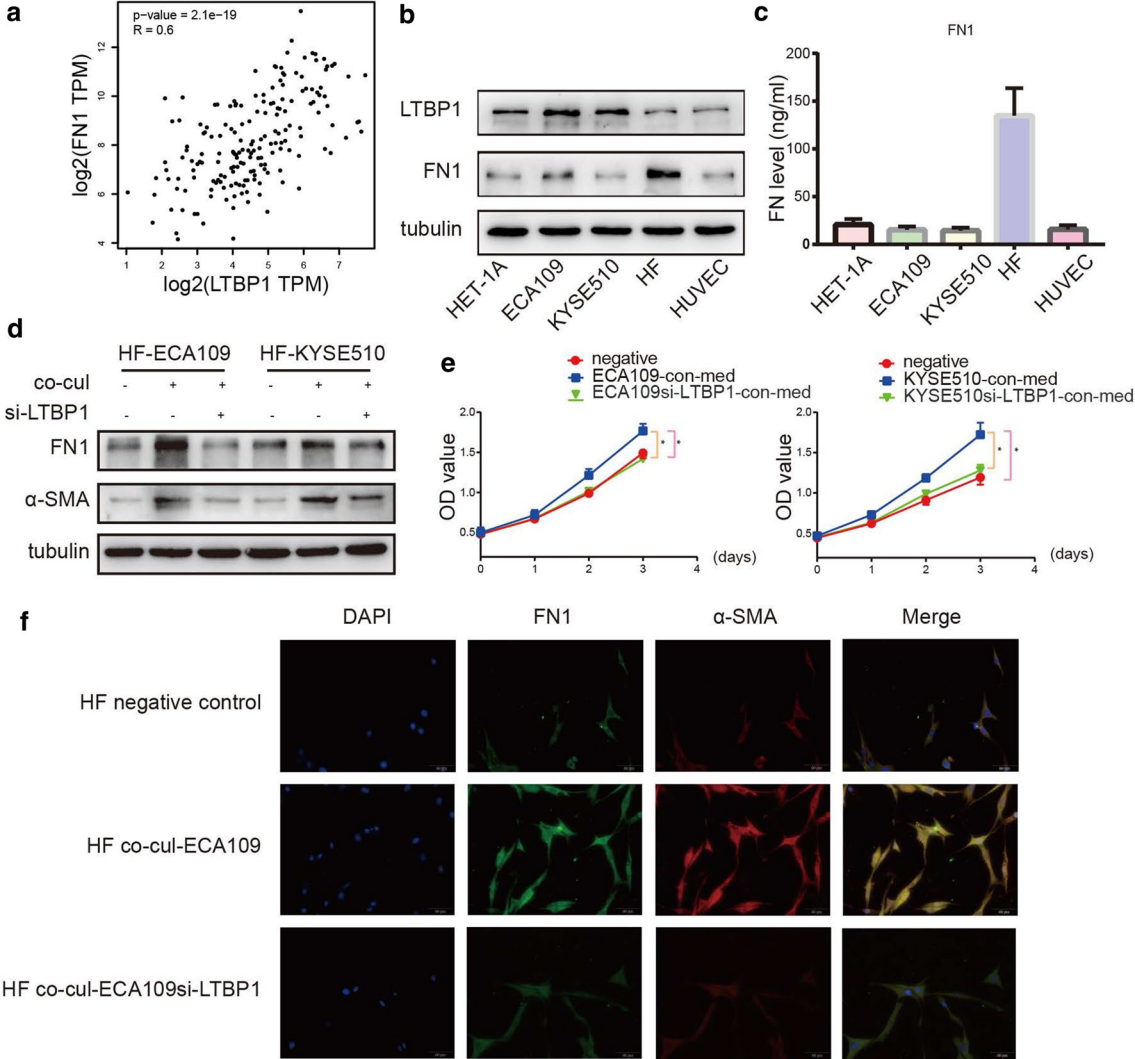
LTBP1 contributed to CAFs transformation and expression of FN1 induced by ESCC cells. **a** The expression of LTBP1 and FN1 were positively correlated in the GEPIA database (Spearman correlation analysis, r = 0.6, p < 0.001). **b**, **c** The expression of LTBP1 and FN1 were detected in various cells by western blot and ELISA. **d**, **f** Western blot and immunofluorescent analysis of α-SMA and FN1 expression in fibroblasts which were con-cultured with si-NC, si-LTBP1 ESCC cells or negative for 96 h. **e** CCK8 assays comparing the effect of (± siLTBP1 ESCC cells or negative) conditioned medium on the activity of the fibroblasts. Each experiment was performed in triplicate. Paired-samples t-test, *p < 0.05

GEPIA dataset (http://gepia.cancer-pku.cn/) was used to explore the correlation of the expression levels of LTBP1 and FN1 in esophageal carcinoma tissues. There was a significant positive correlation between LTBP1 and FN1 expression in esophageal carcinoma (r = 0.6, p < 0.001, spearman correlation analysis) (Fig. 5a). However, the results of Western blot and ELISA indicated that there was a negative relationship between the expression of LTBP1 and FN1 in the same cell lines (Fig. 5b, c), which was inconsistent with the results in GEPIA dataset. The results showed that LTBP1 was highly expressed in ESCC cells but lowly expressed in fibroblasts, while FN1 was highly expressed in fibroblasts but lowly expressed in ESCC cells. Thus, there may be some interactions between the ESCC cells and the adjacent fibroblasts.

To explore the influence of LTBP1 on the interaction between the ESCC cells and the adjacent fibroblasts, we co-cultured HF cells with siNC or siLTBP1 ESCC cells. Primary normal fibroblasts were cultured alone under the same conditions and served as a negative control group. After 96 h of cultivation, the biomarker of the CAF(α-SMA) was assessed. The result showed that the fibroblasts expressed a higher level of α-SMA and FN1 protein in the co-cultured ESCC cells than that in the negative control cells (Fig. 5d, f). SiLTBP1 could reduce α-SMA and FN1 expression in fibroblasts, suggesting that down-regulation of LTBP1 may suppress CAFs transformation. The proliferation activities of the fibroblasts were also validated by CCK8 assays. The results showed that after treatment with ESCC cells condition medium, a higher proliferative activity of the fibroblasts was induced than that in the negative control cells and si-LTBP1 group(Paired-samples t-test, p < 0.05) (Fig. 5e). All the above results suggested that LTBP1 may promote ESCC cell to induce CAFs transformation and more FN1 expression in fibroblasts.

### Correlation between LTBP1 and FN1 expression in ESCC tissue

**Fig. 6:**
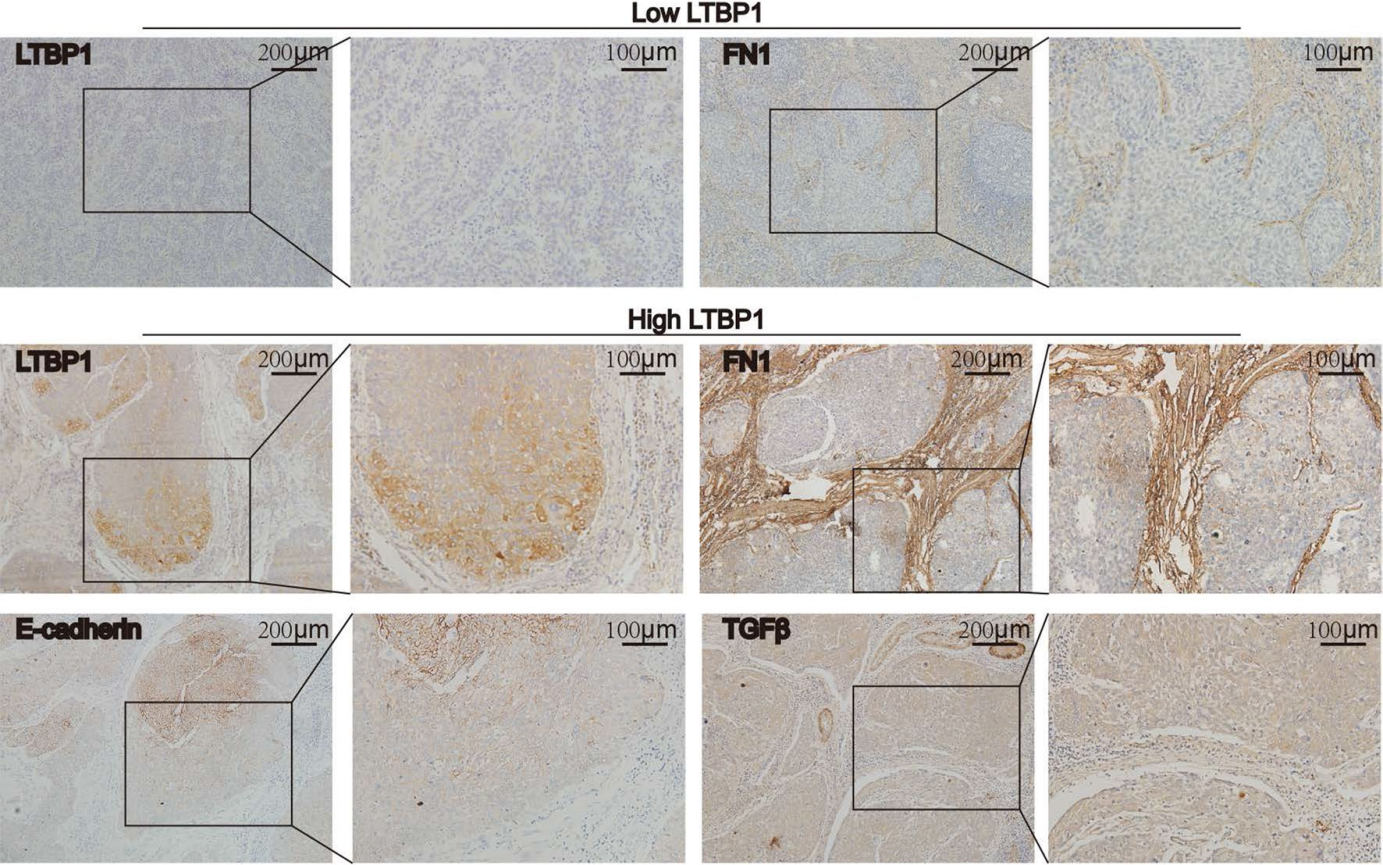
The expression of LTBP1 and FN1 showed positive correlation in ESCC tissues. IHC showed that LTBP1 was mainly observed in cancer cells while FN1 was mainly observed in the surrounding stroma. In LTBP1 positive cases, the expression of LTBP1 was mostly located in tumor parenchymal margin. E-cadherin was highly expressed in tumor parenchymal center and TGFβ was uniformly expressed in tumor parenchyma

We detected the expression of LTBP1 and FN1 in 152 cases of ESCC tissues by IHC. We found that there was a positive relationship between the expression of LTBP1 and FN1(p < 0.001, Chi Square) (Table 2) though they had different localization in ESCC tissues. LTBP1 was mainly observed in cancer cells while FN1 was mainly observed in the surrounding stroma. In LTBP1 positive cases, the expression of LTBP1 was mostly located in tumor parenchymal margin, while E-cadherin was mainly expressed in tumor center (Fig. 6), suggesting that LTBP1 promoted ESCC cells to acquire mesenchymal phenotype in tumor parenchymal margin. Meanwhile, TGFβ was uniformly expressed in the tumor parenchymal (Fig. 6, Additional file 1: Figure S1). Taken together, it was indicated that the overexpression of FN1 is positively associated with the expression of LTBP1, which could also activate the EMT in ESCC.

**Table 2:** Correlation of expression levels of LTBP1 and FN1

	Number	LTBP1 expression		χ^2^	p value
		Low	High		
FN1 expression					
Low	66	57	9	76.298	< 0.001*
High	86	13	73

## Discussion

Esophageal cancer has high mortality and mobility rate and is the sixth leading cause of cancer deaths all of the world. About 80% of cases occur in the developing countries. There is a high prevalence of ESCC in east Asia [1]. Thus, it is urgent to identify molecular markers contributing to ESCC in order to improve early diagnosis and prognosis of ESCC.

In the present study, we used the TMT method to screen DEPs in ESCC tissues and corresponding adjacent tissues. Then 39 up-regulated proteins were screened in ESCC tissue. Some of the DEPs, such as THBS1, LTBP1, PLOD2, RCN3 and FN1, were validated by western blot analyses and GEPIA dataset.

LTBP1 is expressed in various of tissues and cell types [9]. Our study indicated that the expression of LTBP1 was higher in ESCC tissues than that in paired adjacent normal tissues, which is consistent with previous findings in other malignancies [19, 20]. Moreover, we found that high LTBP1 expression was statistically associated with lymph node metastasis in ESCC. The above findings are consistent with the researches of LTBP1 in breast cancer, which also illustrated a positive relationship between high LTBP1 expression and metastasis [23, 27]. In another study, silencing of LTBP1 expression increases cell proliferation in malignant glioma [19]. However, in the present study, no relationships were found between LTBP1 expression and proliferation. Thus, we suggested that LTBP1 may serve as an oncogene during ESCC progression, facilitating lymphatic metastasis of ESCC.

We further explored the functions of LTBP1 in ESCC cells in vivo and in vitro. Our present research indicated that down-regulation of LTBP1 inhibited migratory and invasive properties of ESCC cells in vitro as well as decreased metastatic ability in vivo. We also found that down-regulation of LTBP1 was accompanied by decreased expression of TGFβ, N-cadherin and vimentin, as well as increased expression of E-cadherin in ESCC cells, suggesting that LTBP1 may involve in regulating the secretion and activation of TGFβ and play an important role in promoting EMT in ESCC cells. TGFβ is the most well-known stimulator of EMT and metastasis in cancer [28, 29], so we treated ESCC cells with TGFβ in our study. The results showed that LTBP1 altered the expression of proteins associated with TGFβ-induced EMT. We further found that after stimulation with TGFβ, numerous features of EMT occurred in ESCC cells including morphological changes, loss of epithelial markers and acquisition of mesenchymal markers. Inhibiting expression of LTBP1 reversed the morphological changes and EMT markers induced by TGFβ. EMT is an essential event in tumor development and a trigger of migration, invasion and metastasis in cancer cells since EMT is associated with reduced intercellular adhesion and increased motility [30–32]. Our above studies implied that LTBP1 involved in cancer progression via TGFβ-induced EMT.

In further research, we investigated whether the inhibition of LTBP1 could affect the sensitivity of ESCC cells to 5-Fu treatment. In our study, LTBP1 was a modulator of EMT. Increasing evidences suggest that EMT contributes to chemotherapy resistance in multiple cancer types and may serve as a potential target for overcoming chemoresistance [33]. In this study, we also found that knockdown of LTBP1 also promoted 5-FU-induced apoptosis of ECSS cells, along with the alterations of apoptotic markers, such as the decreased level of BCL-2 and increased level of BAX protein. The results were consistent with previous reports that esophageal cancers cells with EMT growth pattern exhibited more resistance to chemotherapeutic drugs [34, 35]. It has been reported that Anti-EMT properties could promoted apoptosis [36]. As the critical regulators of apoptosis, BCL-2 family proteins are also correlated with chemoresistance [37, 38]. BCL-2 prolongs cell survival after BAX-induced apoptosis [39]. Our work firstly suggested that downregulation of LTBP1 enhanced the effect of 5-FU-induced chemotherapy sensitivity.

In our study, we found the LTBP1 expression showed a significant positive correlation with FN1 expression in ESCA tumors by GEPIA database. However, the expression of LTBP1 and FN1 showed different localization. LTBP1 and FN1 had different expression patterns in tumor cells and fibroblasts. LTBP1 was highly expressed in ESCC cells but lowly expressed in fibroblasts, while FN1 was highly expressed in fibroblasts but lowly expressed in ESCC cells. In LTBP1 highly expressed ESCC tissues, the expression of FN1 was also very high in the stroma surrounding tumor nests. We thought there might be some paracrine interactions between ESCC cells and CAFs transformation. It has been reported that TGFβ is not only known as a stimulator of EMT, but also as a trigger of fibroblasts to achieve myofibroblast phenotype [40, 41]. In our study, we found that silencing LTBP1 in ESCC cells could inhibit CAFs transformation and activity.And silencing LTBP1 in ESCC cells which were co-cultured with fibroblasts can decrease FN1 in fibroblasts. Our findings suggested that LTBP1 may promote CAFs transformation and fibroblasts secretion of FN1 in ESCC. Interestingly, we found that LTBP1 was overexpressed in tumor parenchymal margin. Inversely, E-cadherin was high expressed in tumor center. While as a stimulator of EMT, TGFβ was uniformly expressed in the tumor parenchyma. Thus, we supposed that LTBP1 facilitated TGFβ-induced ESCC cells to acquire mesenchymal phenotype in tumor parenchymal margin. To achieve a better understanding of the mechanism of LTBP1-induced carcinogenesis, further studies are necessary to explore the function of LTBP1 on tumor microenvironment.

## Conclusion

Our findings supported the oncogenic function of LTBP1 in ESCC. Overexpression of LTBP1 was positively associated with lymphatic metastasis in ESCC. Inhibition of LTBP1 decreased ESCC cells invasion and migration capacities and induced EMT changes. Furthermore, LTBP1 involved in TGFβ-induced EMT and affected the progression and chemoresistance of ESCC. LTBP1 promoted ESCC cells to induce CAFs transformation and promoted CAFs to secrete FN1. In general, our study suggested that LTBP1 may be a molecular biomarker of ESCC progression and inhibition of LTBP1 may supply a potential therapy for ESCC.

## Supplementary information

**Additional file 1: Figure S1.** Negative (isotype) controls of TGFβ.

## Data Availability

All data generated or analysed during this study are included in this published article.

## Abbreviations

EMT: Epithelial–mesenchymal transition
CAFs: Cancer-associated fibroblasts
ESCC: Esophageal squamous cell carcinoma
DEPs: Differentially expressed proteins
LTBP1: Latent transforming growth factor β binding protein 1
FN1: Fibronectin 1
ESCA: Esophageal carcinoma
TMT: Tandem mass tag
AJCC: American Joint Committee on Cancer
DMEM: Dulbecco’s modified Eagle’s medium
FBS: Fetal bovine serum
Co-cul: Co-culture
MF: Molecular function
BP: Biological process
CC: Cellular component
PLOD2: Procollagen-lysine,2-oxoglutarate 5-dioxygenase
THBS1: Thrombospondin 1
RCN3: Reticulocalbin 3
IHC: Immunohistochemistry
α-SMA: α-Smooth muscle actin
ELISA: Enzyme linked immunosorbent assay
