# Supplementary figures and images for: LTBP1 promotes esophageal squamous cell carcinoma progression through epithelial-mesenchymal transition and cancer-associated fibroblasts transformation
Source: J Transl Med. 2020 Mar 26;18:139. doi: 10.1186/s12967-020-02310-2 (PMC7098101; doi:10.1186/s12967-020-02310-2)

# Isotype Control

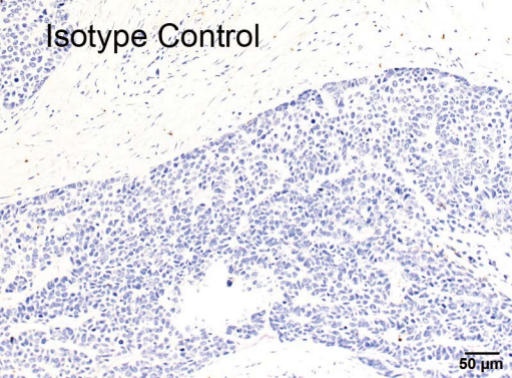

50  $\mu$ m

Supplement: Supplementary file 1 — Additional file 1: Figure S1. Negative (isotype) controls of TGFβ. [file 12967_2020_2310_MOESM1_ESM.pdf]
